# Cultural Relevance and Acceptability of Cognitive Behavioral Therapy Techniques Adapted by AI or a Human Psychologist: Experimental Study

**DOI:** 10.2196/91056

**Published:** 2026-05-04

**Authors:** Youstina Demetry, Per Carlbring, Gerhard Andersson

**Affiliations:** 1 Centre for Psychiatry Research, Department of Clinical Neuroscience, Karolinska Institutet & Stockholm Health Care Services, Norra Stationsgatan 69, Stockholm, 113 64, Sweden, +46 739522332; 2 Department of Psychology, Stockholm University, Stockholm, Sweden; 3 School of Psychology, Korea University, South Korea, Seoul, Republic of Korea; 4 Department of Behavioral Sciences and Learning, Linköping University, Linköping, Östergötland, Sweden; 5 Department of Biomedical and Clinical Sciences, Linköping University, Linköping, Östergötland, Sweden; 6 Department of Health, Education and Technology, Luleå University of Technology, Luleå, Norrbotten, Sweden; 7 HEI-Lab: Digital Human-Environment Interaction Labs, Universidade Lusófona, Lisbon, Portugal

**Keywords:** cognitive behavioral therapy, ethnopsychotherapy, artificial intelligence, AI, cultural adaptation, immigrant, refugee

## Abstract

**Background:**

Evidence-based psychological interventions are usually not accessed by marginalized groups such as refugees. Culturally adapted psychological interventions have reported larger effect sizes than nonadapted psychological interventions. However, the cultural adaptation of interventions is a lengthy process, entailing a challenge. One potential solution to overcome this challenge is the use of artificial intelligence (AI).

**Objective:**

The aim of this study was to investigate and compare the perceived cultural relevance and acceptability of 2 common cognitive behavioral therapy (CBT) techniques when translated and culturally adapted by AI versus a human psychologist.

**Methods:**

In a 2×2 factorial design, the text generator type (AI vs human psychologist) and the CBT technique (cognitive restructuring vs behavior modification) were compared. CBT technique texts translated and culturally adapted either by AI or by a human psychologist were blindly rated using the Cultural Relevance Questionnaire and the Theoretical Framework of Acceptability. Raters were Arabic-speaking refugees and immigrants, aged between 18 and 69 years, residing in Sweden, Denmark, and Germany. Raters were randomly allocated to 1 of 4 conditions. Each condition consisted of 2 stimuli. Two-factor between-subject design analyses were used to analyze the data.

**Results:**

A significant main effect of the text generator domain type (*P*=.02; η²=0.045) was found in the first rating, with texts adapted by the AI domain perceived as more culturally relevant than those adapted by the human domain. No significant main effect of the CBT technique was found in the first rating (*P*=.10; η²=0.022). There were no differences in the second rating. Regarding acceptability, no significant main effects of text generator domain type (*P*=.09; η²=0.024) or the CBT technique (*P*=.88; η²=0.001) were found in either of the ratings.

**Conclusions:**

CBT technique materials adapted by AI may be perceived as similarly culturally relevant as those adapted by a human psychologist. This finding implies the potential to accelerate the cultural adaptation of psychological interventions. However, AI still needs to be used with caution and in accordance with rigorous safety standards and robust frameworks.

## Introduction

### Background

Recently, clinical research targeting refugees and migrants has advocated for culturally adapting evidence-based psychological interventions. Evidence suggests that this leads to slightly larger effect sizes compared to nonadapted psychological interventions and to lower attrition rates. Cultural adaptation considers the cultural values, meanings, and patterns of the individual when developing or adapting psychological interventions [1,2]. One argument against culturally adapting psychological interventions is the lengthy process it entails [3] and that the costs involved may not be worth the outcomes [4]. This leads to the question of whether the process of cultural adaptation could be worthwhile if assisted by artificial intelligence (AI) tools.

In the past few years, interest in using large language models (LLMs)–based generative AI chatbots, such as OpenAI’s ChatGPT, Microsoft’s Copilot, and Anthropic’s Claude, for supporting clinical practices has grown substantially [5]. LLMs are characterized by their capability to use large datasets and to apply natural language processing techniques to generate responses that are complex and contextually relevant [6,7].

In the last year, several methods have been used to integrate LLMs into the diagnosis and treatment of mental disorders [8-10]. One study reported no significant difference between an AI model and psychiatrists in the prediction of depression in a Japanese sample [11]. Another study evaluated an AI-assisted self-referral tool compared to alternative means of referral [12]. The AI-assisted self-referral tool (Limbic Access) generated case presentations and assessments of symptom severity and risk factors such as suicidal ideation. The authors found that accessing care through Limbic Access was associated with reduced clinical assessment time, reduced wait time for assessment, reduced time to treatment, and improved efficacy in service allocation [12]. In another study, Meta’s Llama was trained to identify cognitive distortions, with virtual case examples generated using Claude [13]. In psychotherapy studies, ChatGPT has been used to offer guidance to university students participating in a 4-week intervention targeting perfectionism [14]. Finally, a behavioral activation–based AI chatbot was reported to significantly improve outcomes [15].

Following this global trend, some efforts have been made to evaluate the cultural sensitivity of AI tools in mental health care services. In a recent qualitative study, the researchers aimed to evaluate ChatGPT’s general therapeutic skills and its multicultural counseling skills [16]. ChatGPT was provided with an instruction prompt to take the role of a multicultural therapist, build rapport, and show cultural awareness in its responses. The authors then provided ChatGPT with a series of realistic scenarios containing cultural probes. The authors reported that ChatGPT ignored the prompt and deviated from the instructions given, returning to default, non–culturally sensitive responses. Furthermore, ChatGPT appeared to validate the user’s experience without showing a deeper understanding of it. Consequently, the advice given by ChatGPT was seen as superficial and not particularly culturally sensitive [16]. Finally, the authors noted that ChatGPT lacked self-awareness regarding its own cultural values and patterns. The authors suggested that regardless of the prompt, LLMs rely more heavily on large datasets. This is a problem, as large, culturally specific datasets are scarce. To overcome this, another study trained ChatGPT to generate culturally sensitive questions and descriptions incorporating cognitive behavioral therapy (CBT) elements targeting individuals in China [17]. Overall, the use of generative AI in mental health care is still in its infancy, and this is particularly true for marginalized populations. Furthermore, little is known about how individuals from cultural minority groups in Europe perceive the cultural relevance of AI-generated therapeutic materials.

### Objective of Study

The aim of the study was to investigate and compare the perceived acceptability and cultural relevance of 2 common CBT techniques when translated and culturally adapted using AI versus a human psychologist. More specifically, a behavior modification technique focusing on exposure and a cognitive restructuring technique focusing on the identification of automatic thoughts were translated from Swedish to Arabic and culturally adapted using either an AI domain or a human domain generator.

## Methods

### Study Design

The study used a 2×2 factorial design with 2 trials to allow for the comparison of the text generator domain type (AI vs human) across CBT techniques (cognitive restructuring vs exposure). Raters were randomly assigned to 1 of 4 conditions. Each condition consisted of a combination of 2 of the following four stimuli: (1) exposure exercise culturally adapted by AI domain generator, (2) exposure exercise culturally adapted by a human domain generator, (3) cognitive restructuring exercise culturally adapted by AI domain generator, and (4) cognitive restructuring exercise culturally adapted by a human domain generator. Each participant rated 2 passages. The conditions were counterbalanced (ie, the second rating was always different from the first). The study was preregistered at the Open Science Framework [18]. The CHERRIES (Checklist for Reporting Results of Internet e-Surveys) checklist was used for reporting the results (Checklist 1).

### Ethical Considerations

Ethics approval was not required for this study, as it did not involve procedures falling under the jurisdiction of the Swedish Ethical Review Act (Law 2003:460) [19], that is, it did not involve any physical intervention, any collection of sensitive personal data, or any intention to affect participants physically or psychologically or participants’ identifiable biological materials. Nevertheless, the study adhered to the principles of the Declaration of Helsinki [20]. All raters were informed about the general purpose of the study, the voluntary nature of participation, and their right to withdraw at any time. Informed consent was obtained from all raters prior to data collection (refer to Multimedia Appendix 1 for the informed consent form). Raters were debriefed about the use of AI in generating some of the materials after completing the ratings. Upon completion of the experiment, raters were offered a nonmonetary incentive in the form of points redeemable for gift cards. All data were collected and stored on iTerapi (Vlaescu et al [21]), a secure platform developed at Linköping’s University, which operates in full compliance with the General Data Protection Regulation (GDPR) and ensures strict physical and digital security measures to safeguard confidentiality.

### Power Analysis

Statistical power was estimated based on a previous AI study (Franke Föyen et al [22]) and a clinically relevant difference between the 2 conditions. With an estimated medium effect size of Cohen *d*=0.50 and a desired power of .80, a sample size of 128 raters was required, yielding 32 (25%) raters in each cell of the 2×2 design.

### Raters and Recruitment

Arabic-speaking immigrants and refugees were recruited via personal invitations sent through a data collection company in Europe, Norstat. Thus, the experiment was restricted to invitation-only participation (refer to Multimedia Appendix 2 for the invitation). The invitation stated the voluntary nature of the experiment. Raters were Arabic-speaking refugees or migrant adults aged 18 to 69 years, residing in Sweden, Denmark, and Germany. Second-generation immigrants or refugees were not excluded if they could read and write in Arabic. Recruitment took place between September 25, 2025, and November 12, 2025.

### Procedure and Randomization

A screening question was sent to all panelists asking whether they could comprehend Arabic. Panelists who answered “yes” received an invitation describing the study and its focus on Arabic speakers. Invitations directed potential raters to the study available on Linköping University’s iTerapi platform [21]. Prior to participation in the study, raters provided digital informed consent. Block randomization was used to ensure that each cell had a similar number of raters. The raters were first instructed to read the first passage. They were then asked to rate the passage using the Cultural Relevance Questionnaire (CRQ) [23] and the Theoretical Framework of Acceptability (TFA) [24]. Both questionnaires were presented on 1 page (12 items). The questionnaires were followed by a free-text form where raters could share other thoughts regarding the passage they had read. The raters were then instructed to read a second passage, after which they again completed the CRQ and TFA regarding the second passage. This was also followed by another free-text field where raters could share other thoughts regarding the passage. Thus, each rater read 2 passages and provided a rating per passage using CRQ and TFA. In total, each rater provided 2 ratings.

Afterward, raters were asked to fill out information about demographic characteristics. Then, raters were asked to indicate whether they had any professional experience with psychological interventions and whether they had any assumptions regarding how the passages had been produced. Answering all items was mandatory to move forward to the next page. Finally, raters were debriefed. The experiment was presented in 9 pages. Raters were able to review or change their responses using a back button.

### The 2 CBT Texts

Two CBT texts were extracted from existing treatment modules available on Linköping University’s secure treatment platform, iTerapi [21]. The techniques were (1) exposure for anxiety reduction and (2) identification of automatic thoughts for cognitive restructuring. The 2 treatment modules were derived from existing Swedish treatment modules used in studies on internet-delivered CBT (iCBT). Evidence on the efficacy of the treatment modules and detailed descriptions of these modules are reported elsewhere [25,26].

### The Prompt

Generative AI was prompted using a role-adoption, constraint-based framework. The generative AI was prompted to assume the role of a licensed psychologist with specialist expertise in clinical psychology and cultural adaptation of iCBT. The prompt was further constrained by the 8 domains of the ecological validity framework for cultural adaptation by Bernal et al [1]. The framework was originally developed for Hispanic populations and consists of eight domains: (1) language, (2) persons, (3) metaphors, (4) content, (5) concepts, (6) goals, (7) methods, and (8) context [1]. Its use has expanded to psychological interventions targeting other ethnic minority groups, such as Japanese women with bulimia nervosa [27] and Latinx youth and Colombian students with depression [23,28]. Furthermore, it has been used to facilitate the cultural adaptation of a support program targeting Swiss caregivers of individuals with dementia [29]. The command was executed on June 23, 2025. LLM agents were prompted. The prompt is available in Multimedia Appendix 3.

### The Human Domain Generator

The human domain generator, a psychologist with expertise in the cultural adaptation of psychological interventions, received the same prompt, excluding the role-adoption command. The non–culturally adapted materials were sent to the human domain generator on June 25, 2025, and the culturally adapted materials were returned on June 30, 2025. It took the human domain generator 4.5 hours to complete the task, of which 2 hours were devoted to reviewing and proofreading the materials.

The human domain generator was a clinical psychologist with a Syrian background residing in Sweden and fluent in Swedish. He received his Master’s in psychology from Damascus University in 2013. He relocated to Sweden and completed a program for psychologists with foreign degrees at Uppsala University in 2025. He has been working in the research field of mental health and psychosocial support for individuals with a migration background since 2016 and has experience with the cultural adaptation of iCBT material, but not with the modules used in this study.

### The Generated Cultural Adaptation of the Stimuli Materials

The texts for both cognitive restructuring and exposure were processed using 2 known LLM agencies. For the materials generated by the AI and the human psychologists, refer to Multimedia Appendix 4.

### Instruments

#### Cultural Relevance

The CRQ aims to assess the degree of cultural relevance of psychological interventions [23]. The original version of CRQ consists of 2 sections. The first section assesses the psychological intervention as a whole, while the second section assesses the cultural relevance of individual modules. For the purpose of this study, only the first section was used.

The first section comprises 3 items: functional equivalence, conceptual equivalence, and linguistic equivalence. The functional equivalence consists of the following three statements: (1) “The programme involves behavioral or emotional expressions familiar to the cultural group being targeted,” (2) “the people and cultural context are reflected in the programme (e.g. social, political, economic, ethnic, historical),” and (3) “the programme goals are tailored to work with the user from this cultural context (e.g. examples, personal stories).” The conceptual equivalence consists of 1 statement: “The treatment includes symbols and concepts shared by the cultural group, for instance cultural expressions of depression and anxiety, ideas or analogies about mental illness are included in the program.” Finally, the linguistic equivalence consists of 1 statement: “the treatment includes written and oral communication that can be considered dialects and jargon relevant in this cultural context (e.g. regionalism, slang).”

All statements were rated on a 5-point Likert scale ranging from 1 (“the components are not reflected within the programme”) to 5 (“All of the components are reflected within the programme”). For the purpose of this study, the terms “programme” and “treatment” were substituted with “exercise.” In this study, the CRQ had an internal consistency of α=.78.

#### Acceptability

The TFA [24] was used to assess the degree of acceptability. The TFA consists of 8 items. The first 7 items assess affective attitude, burden, ethicality, perceived effectiveness, intervention coherence, self-efficacy, and opportunity costs. The final item assesses the general acceptability of the intervention.

The first item asks, “did you like or dislike [the intervention]?” and is rated on a 5-point Likert scale ranging from 1 (“strongly dislike”) to 5 (“strongly like”). The second item asks, “how much effort did it take to read through [the intervention]?” and is rated on a 5-point Likert scale ranging from 1 (“no effort at all”) to 5 (“huge effort”). The third item states “there would be a moral or ethical consequence of conducting [the behavior]” and is rated on a 5-point Likert scale ranging from 1 (“strongly disagree”) to 5 (“strongly agree”). The fourth item states “the [intervention] improves my [behavior/condition/clinical outcome]” and is rated on a 5-point Likert scale ranging from 1 (“strongly disagree”) to 5 (“strongly agree”). The fifth item states “it is clear to me how [the intervention] will help [manage/improve] my [behavior/condition/clinical outcome]” and is rated on a 5-point Likert scale ranging from 1 (“strongly disagree”) to 5 (“strongly agree”). The sixth item asks, “how confident did you feel about [behavior i.e. engaging with] [the intervention]?” and is rated on a 5-point Likert scale ranging from 1 (“very unconfident”) to 5 (“very confident”). The seventh item states “(behavior i.e. engaging in) [the intervention] interfered with my other priorities” and is rated on a 5-point Likert scale ranging from 1 (“strongly disagree”) to 5 (“strongly agree”). The final, general acceptability item asks, “how acceptable was the [intervention] to you?” and is rated on a 5-point Likert scale ranging from 1 (“completely unacceptable”) to 5 (“completely acceptable”). A recent study has evaluated the TFA face validity in a Swedish context [30]. In this study, the TFA had an internal consistency of α=.60.

#### Knowledge of Psychological Interventions

To assess raters’ knowledge of psychological interventions, the research team developed a yes-or-no question stating, “Are you currently working or have you previously worked with psychological interventions?”

#### Production of Extracted Texts

To assess whether raters held any presumptions regarding how the texts were produced, the research team developed the following open-ended question: “Did you have any idea how the passages were produced?”

### Statistical Analyses

Data analyses were performed using SPSS (version 31; IBM Corp). The primary analysis was a 2-factor between-subjects ANOVA to evaluate the main effects of the text generator domain type (AI vs human) and the CBT technique (exposure vs identification of automatic thoughts), as well as their interaction on the ratings of perceived cultural relevance. In another model, effects on ratings of acceptability were tested. Model assumptions were assessed by examining residual normality using Q-Q plots and the Shapiro-Wilk test. The Levene test was used to assess the homogeneity of variances. Multiple regression models were conducted to account for potential covariates. The covariates included professional experience in mental health (yes or no), age, gender, and country of birth. Assumptions about whether the materials were AI-generated or not were analyzed descriptively.

## Results

### Characteristics of the Raters

A sample of 581 raters completed the experiment; however, 453 (78%) were excluded for either not being Arabic-speaking immigrants or refugees or for having completed the experiment in less than 10 minutes. The view rate, defined as the ratio of unique survey visitors to unique site visitors, was 0.21. The completion rate, defined as the ratio of visitors who completed the experiment to those who agreed to participate, was 2.36. The final sample consisted of 128 raters. In total, 1 (0.8%) participant reported relocating from Denmark to Iraq. Approximately two-thirds of the participants were females (n=89, 69.5%). Approximately one-third of the sample was second-generation immigrants or refugees. Table 1 summarizes the characteristics of the sample.

**Table 1.** Demographics of the raters (N=128).

Characteristics	Participants
Age (years)	
Mean (SD)	38.55 (14)
Range	18-69
Sex, n (%)	
Male	39 (30.5)
Female	89 (69.5)
Country of birth, n (%)	
Syria	30 (23.4)
Iraq	27 (21.1)
Sweden	23 (18.0)
Denmark	15 (11.7)
Lebanon	12 (9.4)
Palestine	5 (3.9)
Germany	4 (3.1)
Morocco	3 (2.3)
Algeria	2 (1.6)
Egypt	2 (1.6)
Jordan	2 (1.6)
Kurdistan	1 (0.8)
Tunisia	1 (0.8)
Saudi Arabia	1 (0.8)
Country of residence, n (%)	
Sweden	80 (62.5)
Denmark	33 (25.8)
Germany	13 (10.2)
Switzerland	1 (0.8)
Iraq	1 (0.8)
Parents‘ country of birth^a^, n (%)	
Iraq	12 (9.4)
Lebanon	11 (8.6)
Syria	6 (4.7)
Palestine	6 (4.7)
Kuwait	2 (1.6)
Morocco	1 (0.8)
Egypt	1 (0.8)
Yemen	1 (0.8)
Tunisia	1 (0.8)
Highest education level, n (%)	
No formal education	1 (0.8)
Primary education	2 (1.6)
Secondary education	29 (22.6)
Bachelor’s degree	59 (46.1)
Master’s degree	31 (24.2)
Doctoral degree	5 (3.9)
Other	1 (0.8)
Employment status, n (%)	
Full-time employment	66 (51.6)
Part-time employment	16 (12.5)
Unemployed	7 (5.5)
Self-employed	8 (6.2)
Student	26 (20.3)
Retired	4 (3.1)
Other	1 (0.8)
Works in the field of psychological interventions, n (%)	
No	109 (85.2)
Yes	19 (14.8)

a Includes only second-generation immigrants.

### Cultural Relevance

Table 2 includes a summary of the descriptive statistics for the cultural relevance scores from the ratings. The table presents 4 different variables. The first variable is the sum score of the CRQ. The subsequent 3 variables are the subscales of the cultural relevance measurement, comprising the functional equivalence, the conceptual equivalence, and the linguistic equivalence.

**Table 2.** Descriptive statistics summarizing the ratings of the cultural relevance.

Study conditions	Mean (SD)
First rating	
Cultural relevance	
AI^a^	
Exposure	18.19 (4.53)
Cognitive restructure	18.81 (3.02)
Human	
Exposure	15.97 (4.33)
Cognitive restructure	16.81 (3.71)
Functional equivalence	
AI	
Exposure	3.57 (1.02)
Cognitive restructure	3.76 (0.60)
Human	
Exposure	3.13 (0.98)
Cognitive restructure	3.58 (0.93)
Conceptual equivalence	
AI	
Exposure	3.81 (1.12)
Cognitive restructure	3.78 (1.01)
Human	
Exposure	3.16 (1.14)
Cognitive restructure	3.59 (0.84)
Linguistic equivalence	
AI	
Exposure	3.56 (1.16)
Cognitive restructure	3.50 (0.98)
Human	
Exposure	3.03 (1.13)
Cognitive restructure	3.66 (1.19)
Second rating	
Cultural relevance	
AI	
Exposure	18.78 (3.83)
Cognitive restructure	17.59 (5.38)
Human	
Exposure	17.41 (3.81)
Cognitive restructure	18.44 (3.44)
Functional equivalence	
AI	
Exposure	3.73 (0.81)
Cognitive restructure	3.46 (1.12)
Human	
Exposure	3.45 (0.76)
Cognitive restructure	3.67 (0.73)
Conceptual equivalence	
AI	
Exposure	3.78 (0.98)
Cognitive restructure	3.69 (1.23)
Human	
Exposure	3.47 (1.05)
Cognitive restructure	3.78 (0.94)
Linguistic equivalence	
AI	
Exposure	3.69 (1.20)
Cognitive restructure	3.31 (1.42)
Human	
Exposure	3.50 (0.92)
Cognitive restructure	3.31 (1.42)

a AI: artificial intelligence.

A 2-way between-subjects ANOVA was conducted to examine the effects of the text generator domain type (AI vs human) and CBT technique (exposure vs identification of automatic thoughts) on cultural relevance. The Levene test indicated that the assumption of homogeneity of variance was met (*F*_3,124_=1.37; *P*=.25). The Shapiro-Wilk test indicated that the assumption of normality of variance was met (Shapiro-Wilk W=0.99; *P*=.33).

For the first rating, a significant main effect of text generator domain type (*F*_1,127_=5.86; *P*=.02; η²=0.045) was found, with AI-generated texts receiving higher scores than human-adapted text. The main effect of the CBT technique was not statistically significant (*F*_1,127_=2.75; *P*=.10; η²=0.022). Likewise, the interaction between the text generator domain type and the CBT technique was not significant (*F*_1,127_=0.58; *P*=.45; η²=0.005).

For the second rating, the Levene test indicated that the assumption of homogeneity of variance was met (*F*_3,124_=2.50; *P*=.06). The Shapiro-Wilk test indicated that the assumption of normality of variance was violated (W=0.97; *P*=.004). However, visual inspection of the Q-Q plot showed no substantial deviations from normality (Multimedia Appendix 4). For the second rating, there was no significant main effect of text generator domain type (*F*_1,127_=0.13; *P*=.72; η²=0.001) and no significant main effect of the CBT technique (*F*_1,127_=0.011; *P*=.92; η²=0.001). Likewise, the interaction between the text generator domain type and the CBT technique was not significant (*F*_1,127_=2.25; *P*=.14; η²=0.018). Table 3 summarizes the findings of the CRQ subscales.

**Table 3.** Results of 2-factor ANOVA regarding the cultural relevance of cognitive behavioral therapy (CBT) texts.

Variables	*F* value (*df*)	*P* value	η²
First rating			
Text generator domain type			
Functional equivalence	4.11 (1)	.05	0.03
Conceptual equivalence	4.34 (1)	.02	0.04
Linguistic equivalence	0.90 (1)	.34	0.01
CBT technique			
Functional equivalence	4.39 (1)	.04	0.03
Conceptual equivalence	1.24 (1)	.27	0.01
Linguistic equivalence	2.04 (1)	.16	0.02
Text generator domain type×CBT technique			
Functional equivalence	0.77 (1)	.38	0.01
Conceptual equivalence	1.65 (1)	.20	0.01
Linguistic equivalence	3.04 (1)	.08	0.02
Second rating			
Text generator domain type			
Functional equivalence	0.06 (1)	.81	0.00
Conceptual equivalence	0.49 (1)	.48	0.00
Linguistic equivalence	0.23 (1)	.88	0.00
CBT technique			
Functional equivalence	0.03 (1)	.87	0.00
Conceptual equivalence	1.51 (1)	.22	0.01
Linguistic equivalence	1.17 (1)	.29	0.01
Text generator domain type×CBT technique			
Functional equivalence	2.55 (1)	.11	0.02
Conceptual equivalence	1.11 (1)	.29	0.01
Linguistic equivalence	0.82 (1)	.37	0.01

### Acceptability

Table 4 includes a summary of the descriptive statistics for the acceptability scores from the ratings.

**Table 4.** Mean (SD) acceptability scores by study condition.

Variables	Mean (SD)
First rating	
AI^a^	
Exposure	3.50 (0.46)
Cognitive restructuring	3.45 (0.53)
Human	
Exposure	3.30 (0.55)
Cognitive restructuring	3.31 (0.53)
Second rating	
AI	
Exposure	3.39 (0.51)
Cognitive restructuring	3.38 (0.63)
Human	
Exposure	3.45 (0.40)
Cognitive restructuring	3.37 (0.37)

a AI: artificial intelligence.

A 2-way between-subjects ANOVA was conducted to examine the effects of the text generator domain type (AI vs human) and CBT technique (exposure vs identification of automatic thoughts) on acceptability. The Levene test indicated that the assumption of homogeneity of variance was met (*F*_3,124_=0.181; *P*=.91). The Shapiro-Wilk test indicated that the assumption of normality of variance was violated (W=0.97; *P*=.002). However, visual inspection of the Q-Q plot showed no substantial deviations from normality (Multimedia Appendix 4). For the first rating, there was no significant main effect of the text generator domain type (*F*_1,127_=2.99; *P*=.09; η²=0.024) and no effect of the CBT technique (*F*_1,127_=0.023; *P*=.88; η²=0). Likewise, the interaction between the text generator domain type and the CBT technique was found to be not significant (*F*_1,127_=0.201; *P*=.66; η²=0.002).

For the second rating, the Levene test indicated that the assumption of homogeneity of variance was met (*F*_3,124_=1.09; *P*=.36). The Shapiro-Wilk test indicated that the assumption of normality of variance was violated (W=0.96; *P*<.001). However, visual inspection of the Q-Q plot showed no substantial deviations from normality (Multimedia Appendix 5). For the second rating, again, there was no significant main effect of text generator domain type (*F*_1,127_=0.005; *P*=.95; η²=0.0001). No significant main effect of the CBT technique was found (*F*_1,127_=0.431; *P*=.51; η²=0.003). Likewise, the interaction between the text generator domain type and the CBT technique was not statistically significant (*F*_1,127_=0.32; *P*=.57; η²=0.003).

## Discussion

### Principal Findings

This study compared the perceived acceptability and cultural relevance of translated and culturally adapted CBT texts provided by an LLM conversational agent to those provided by a human domain expert. The results indicate that the perception of the cultural relevance and acceptability of AI-generated, culturally adapted CBT materials was comparable to that of materials generated by a human psychologist; however, the AI-generated texts were rated higher than the text generated by the human domain expert on perceived cultural relevance. Furthermore, raters were unable to identify how the therapeutic materials had been generated. This is a crucial finding, as recent research demonstrates that the perceived source matters; specifically, responses attributed to humans are often rated as more empathic and supportive than those attributed to AI, even when the content is identical [31]. In terms of acceptability, AI was not found to be superior to the human domain generator. This finding is not surprising, as generative AI is known for its validating and supportive tone [32], to the extent that there is ongoing debate about whether it may reinforce psychotic thinking [33]. Furthermore, recent commentary argues that treating AI as a trustworthy partner risks increasing anthropomorphization; instead, the focus should be on ensuring the system’s strict adherence to ethical principles such as nonmaleficence and transparency [34].

### Strengths

This study offers some valuable insights. First, the study targets a marginalized and hard-to-reach group in Europe. This is in line with the Diagnostic Statistical Manual of Mental Disorders’ recognition of the role that culture plays in the etiology and expression of mental disorders [35]. Furthermore, the study contributes to the novel discussion on the use of AI tools in culturally adapted CBT. The use of the CRQ and TFA contributed to an improved construct validity in this study.

### Limitations

There are some limitations that should be mentioned. The human domain generator condition was represented by a single generator; therefore, it does not capture the variability between human generators. However, this mimics a real-life setting, where cultural adaptation is usually carried out by 1 person. That said, cultural adaptation in real-world settings often involves multiple rounds of iterative refinement. Cultural adaptation, per se, is a complex task, and using standard LLMs for this purpose constitutes a nonlinear process determined by various factors, including the prompt. Therefore, the findings should be interpreted as specific to the human domain generator and the standard LLM adaptation outputs examined in this study, which may limit their generalizability to other generators and workflows. Furthermore, the results should be interpreted as a “spot-check” of the perceived cultural relevance and acceptability of the output materials. The study did not target how actual patients would rate the material, and it would be useful to examine how individuals in need of treatment perceive these CBT texts. The study is strongly influenced by both the skills of the human translator and the AI system used, as in particular, the latter is a moving target, and Arabic is a large language for AI to derive information from. However, the quality of Arabic is still negotiable in comparison to English. Third, although there are some indications that cultural adaptation makes a difference, it is also hard to include all important factors; for example, political and religious factors can divide populations and hence be sensitive to include in the adaptation process. Therefore, AI tools should be designed to exhibit cultural sensitivity rather than cultural specificity.

### Conclusions

The results of this study provide some evidence for AI’s ability to accelerate the cultural adaptation process of psychological interventions. This could lower the costs of developing culturally adapted psychological interventions and facilitate access to such evidence-based psychological interventions. However, to ensure innovation does not bypass rigorous safety standards, the future implementation of such AI tools must be guided by robust frameworks such as TEQUILA (trust, evidence, quality, usability, interests, liability, and accreditation) [36], ensuring that efficiency does not supersede trust, evidence, and quality. Methods such as retrieval-augmented generation could be tested in future research to test AI’s ability to culturally adapt evidence-based interventions. The study is also relevant for text-based health information in general, as there is a need for multilingual health information in many clinical settings, including Sweden.

## Supplementary material

10.2196/91056 Multimedia Appendix 1 Informed consent.

10.2196/91056 Multimedia Appendix 2 Invitation.

10.2196/91056 Multimedia Appendix 3 The prompt.

10.2196/91056 Multimedia Appendix 4 The generated therapeutic materials.

10.2196/91056 Multimedia Appendix 5 The Q-Q plots.

10.2196/91056 Checklist 1 CHERRIES checklist.

## Abbreviations

AI: artificial intelligence
CBT: cognitive behavioral therapy
CHERRIES: Checklist for Reporting Results of Internet e-Surveys
CRQ: Cultural Relevance Questionnaire
GDPR: General Data Protection Regulation
iCBT: internet-delivered cognitive behavioral therapy
LLM: large language model
TEQUILA: trust, evidence, quality, usability, interests, liability, and accreditation
TFA: Theoretical Framework of Acceptability
