# Supplementary material for: Cultural Relevance and Acceptability of Cognitive Behavioral Therapy Techniques Adapted by AI or a Human Psychologist: Experimental Study
Source: JMIR Form Res. 2026 May 4;10:e91056. doi: 10.2196/91056 (PMC13138788; doi:10.2196/91056)
Supplement: Multimedia Appendix 1 [file formative-v10-e91056-s001.docx]

**Appendix 1: Consent Form**

**Perception of Culturally Adapted Psychological Interventions**

Formulärets överkant

Formulärets nederkant

We are interested in exploring how psychological interventions are perceived when adapted to fit Arabic-speaking individuals. The study is presented by Karolinska Institute and Linköping’s University in Sweden. You will be asked to read passages and answer questions, so please sit comfortably with a mobile phone, a tablet or a laptop.

For questions feel free to get in touch:

**PRINCIPAL INVESTIGATOR** 
Professor Gerhard Andersson
Department of Behavioral Sciences and Learning; Division of Psychology
Linköping University
+4613285840
[Gerhard.andersson@liu.se](mailto:Gerhard.andersson@liu.se)

**CONTACT PERSON**
Youstina Demetry
Centre for Psychiatry Research, Institution of Clinical Neuroscience
Karolinska Institute
+46702542709
[Youstina.demetry@ki.se](mailto:Youstina.demetry@ki.se)

In the coming pages you will be read passages and answers a number of questions. You will firstly be presented with a passage to read. The passage presents a technique used in psychological interventions. You will then be asked to answer some questions to indicate how you perceived the passage.

You will then be asked to read another passage, followed by more questions about the passage.

Since we are interested in knowing what different individuals think of the passages, you will be asked to answer a few questions about yourself.

It will take approximately 20 minutes to complete the tasks

**CONFIDENTIALITY**

Your responses to this survey will be anonymous. Your responses and results will be processed in a way that prevents unauthorized access.

**VOLUNTARY PARTICIPATION**

Your participation in this study is voluntary. It is up to you to decide whether or not to take part in this study. If you decide to take part in this study, you will be asked to tick a box below. After you sign the consent form, you are still free to withdraw at any time and without giving a reason.

**CONSENT**

I have read and I understand the provided information. I understand that my participation is voluntary and that I am free to withdraw at any time, without giving a reason and without cost.

- I voluntarily agree to take part in this study.
