# Supplementary material for: Cultural Relevance and Acceptability of Cognitive Behavioral Therapy Techniques Adapted by AI or a Human Psychologist: Experimental Study
Source: JMIR Form Res. 2026 May 4;10:e91056. doi: 10.2196/91056 (PMC13138788; doi:10.2196/91056)
Supplement: Multimedia Appendix 2 [file formative-v10-e91056-s002.docx]

**Uppfattning av kulturanpassade psykologiska insatser**

Vi är intresserade av att undersöka hur psykologiska interventioner uppfattas när de anpassas för **arabisktalande personer**. Studien presenteras av Karolinska Institutet och Linköpings universitet. Du kommer att bli ombedd att läsa texter som presenterar olika tekniker som används i psykologiska interventioner och svara på frågor. **Undersökningen riktar sig till dig som behärskar arabiska i läs och skrift.** Det tar ungefär 20 minuter att genomföra uppgifterna.

**إدراك التدخلات النفسية الملائمة للثقافة**

نحن مهتمون بدراسة كيفية تصور التدخلات النفسية عندما يتم تكييفها لتناسب **الأشخاص الناطقين باللغة العربية**. يتم تقديم الدراسة من قبل معهد كارولينسكا وجامعة لينشوبينغ. سيُطلب منك قراءة نصوص تعرض تقنيات مختلفة مستخدمة في التدخلات النفسية والإجابة على أسئلة. **تستهدف الدراسة الأشخاص الذين يجيدون القراءة والكتابة باللغة العربية**. يستغرق إكمال المهام حوالي 20 دقيقة.

**Opfattelse af kulturelt tilpassede psykologiske interventioner**

Vi er interesserede i at undersøge, hvordan psykologiske interventioner opfattes, når de er tilpasset **arabisktalende personer**. Undersøgelsen præsenteres af Karolinska Institutet og Linköpings Universitet. Du vil blive bedt om at læse tekster **på arabisk**, der præsenterer forskellige teknikker, der anvendes i psykologiske interventioner, og besvare spørgsmål. **Undersøgelsen er rettet mod dem, der behersker flydende arabisk i læsning og skrivning**. Det vil tage cirka 20 minutter at gennemføre opgaverne.

**Wahrnehmung kulturell angepasster psychologischer Interventionen**

Wir untersuchen, wie psychologische Interventionen wahrgenommen werden, wenn sie für **arabischsprachige** Menschen angepasst sind. Die Studie wird vom Karolinska Institutet und der Universität Linköping durchgeführt. Sie werden gebeten, Texte zu verschiedenen Techniken psychologischer Interventionen zu lesen und Fragen zu beantworten. **Die Umfrage richtet sich an Personen mit fließenden Arabischkenntnissen**. Die Bearbeitung der Aufgaben dauert etwa 20 Minuten.
