# Supplementary material for: Cultural Relevance and Acceptability of Cognitive Behavioral Therapy Techniques Adapted by AI or a Human Psychologist: Experimental Study
Source: JMIR Form Res. 2026 May 4;10:e91056. doi: 10.2196/91056 (PMC13138788; doi:10.2196/91056)
Supplement: Multimedia Appendix 3 [file formative-v10-e91056-s003.docx]

**Appendix 2: The prompt**

Assume the role of a licensed psychologist with specialist expertise in clinical psychology and cultural adaptation of internet-based cognitive behavioral therapy. Translate the text from Swedish to Arabic. The text should be adapted for Syrians between 18–65 years old who have migrated from their home country. Avoid the use of Latin words, phrases, and letters. When translating from Swedish to Arabic, follow these guidelines to ensure a culturally appropriate adaptation based on Bernal, Bonilla, and Bellido’s eight dimensions of cultural sensitivity:

1. Language Dimension: Go beyond mechanical translation and use culturally grounded language Adapt linguistic nuances to capture emotional expressions in Arabic. Be attentive to dialectal variations when needed. Consider the use of Modern Standard Arabic versus Levantine Arabic when appropriate. Use natural expressions that sound familiar to Arabic speakers
2. Persons Dimension: Consider how cultural similarities and differences affect the communication relationship. Adapt address and level of formality to the Arabic cultural context Take hierarchical relationships into account where relevant. Consider family dynamics and any changes in them resulting from migration.
3. Metaphor Dimension: Include symbols and concepts shared by the target population. Replace Swedish proverbs, idioms, and metaphors with equivalent Arabic expressions. Ensure that the proverbs, idioms, and metaphors are rooted in the culture. Use *amthal* (Arabic proverbs) where suitable. Integrate cultural aspects and Arabic proverbs into the text. To increase the relevance of a proverb, add a reflection after it
4. Content Dimension: Integrate knowledge of Arabic values, traditions, and customs. Consider unique social, economic, historical, and political factors. Be aware of regional differences within the Arabic-speaking world. Validate group-specific experiences and perspectives
5. Concept Dimension: Ensure that the concepts addressed are compatible with Arabic culture and context. Take collectivist versus individualist perspectives into account. Balance universal (etic) and culture-specific (emic) aspects
6. Goal Dimension: Frame goals in line with Arabic values and traditions. Support the transmission of positive and adaptive cultural values. Respect key values such as family cohesion, respect, and honor.
7. Method Dimension: Adapt methods and examples to be culturally recognizable. Consider including family perspectives where relevant. Use cultural reinterpretation to explain complex concepts
8. Context Dimension: Take into account changing contexts in Arabic-speaking regions. Consider differences between urban and rural areas. Be mindful of spiritual and social contexts. Consider economic and political factors that may influence interpretation. Take into account aspects of migration and/or the refugee journey, as well as post-migration factors. Consider intergenerational differences (e.g., between first- and second-generation immigrants).

Finally, ensure that the translated text conveys the original content and is ecologically valid—that is, that it is experienced in the intended way within the Arabic cultural context. Cultural aspects should be integrated directly into the main text. For attached figures, images, and tables, ensure that captions, labels, and other text components are also translated and culturally adapted according to the above guidelines.
