# Supplementary material for: Cultural Relevance and Acceptability of Cognitive Behavioral Therapy Techniques Adapted by AI or a Human Psychologist: Experimental Study
Source: JMIR Form Res. 2026 May 4;10:e91056. doi: 10.2196/91056 (PMC13138788; doi:10.2196/91056)
Supplement: Multimedia Appendix 4 [file formative-v10-e91056-s004.docx]

**The Generated Culturally Adapted CBT Techniques by both AI and a Human Expert**

**Stimulus 1: AI – exposure**

**القلق: جرس إنذار الجسد**
قد يعاني بعضنا من قلقٍ شديدٍ يتكرّر أو يدوم طويلاً، أو من نوباتٍ قصيرةٍ تزول سريعاً. يُعدّ القلق استجابةَ جسدك عندما يبدو أمرٌ ما خطِراً؛ فهو شكلٌ من أشكال الخوف وجزءٌ من نظام البقاء. في الماضي البعيد، أنقذ القلق الإنسانَ من الحيوانات المفترسة أو منحه قوةً عند مواجهة الأخطار. عندما رأى أجدادُنا أسداً كانت قلوبهم تخفق بسرعة وتضطرب أفكارهم. لهذا يكون القلق قوياً؛ لأنّه يضع الجسد كله في حالة استعداد للقتال أو الفرار. وكما يقول المثل العربي: «مَن خافَ سَلِم»؛ أي إنّ الخوف قد يَحفظ النفس، لكن علينا إدارة خوفنا بدلاً من السماح له بالتحكّم بنا.

اليوم ما زلنا نشعر بالاستجابة نفسها عند استشعار الخطر، سواء أكان حقيقياً كالتعرّض لسيارة مسرعة أم فكرياً كفكرة «لن يتحدّث أحدٌ معي». الجسد لا يفرِّق بين الخطر الواقعي والمتخيَّل، فتكون ردّة فعله واحدة. ومهما اشتدّ القلق فإنه في ذاته غيرُ ضارّ؛ لأنّه جزءٌ من نظام البقاء، وليس عدواً لك بل استجابة تريد حمايتك.

**كيف تُخفِّف من القلق**

المشكلة ليست الموقف بحدّ ذاته بل استجابة القلق. ولتقليلها عليك أن تفعل عكس ما يمليه عليك حدسك: ابقَ في الموقف بدلاً من الهرب. تُظهر الدراسات أنّ القلق يبلغ الذروة ثم يبدأ بالانخفاض تلقائياً إذا لم تفرّ. إنْ بقيتَ بالوضع المثير للقلق قد يستغرق الانخفاض وقتاً أطول، لكنه سيحدث على أيّ حال. يُسمّى هذا «منحنى القلق»؛ إذ يرتفع ثم يهبط. تذكّر قولنا الشامي: «بعد الضيق الفرج» أي إنّ الشدّة لا تدوم.

**
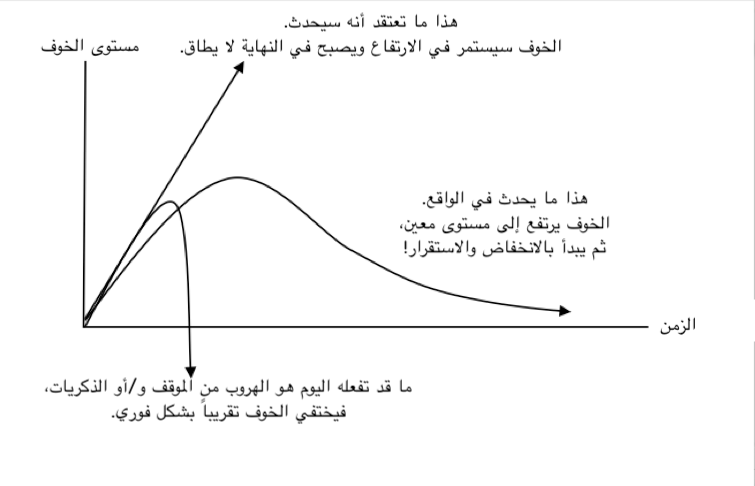
منحنى القلق**

**عن التعرّض التدريجي**

التعرّض التدريجي أحدُ أكثر أساليب العلاجِ المعرفيِّ السلوكيِّ فاعليةً، سواء كنتَ تخاف الكلاب، أو التحدّث أمام الناس, أو المستقبل، أو الانهيار. فكرته أن تعوِّد نفسك تدريجياً على ما يثير قلقك؛ قد يكون غير مريحٍ لكنه فعّال. ومهما بلغت شدة القلق فإنه ينخفض في النهاية—حقيقةٌ فسيولوجية. وكلما واجهت القلق بجرعاتٍ مناسبة وبتكرارٍ كافٍ قلّ تأثيره في المرات التالية.

"الصبر مفتاح الفرج" - تذكيرٌ بأنّ المثابرة في التعرّض تؤتي ثمارها مع الزمن

**تمرين: سُلَّم القلق**

نودّ التعرّف أكثر إلى قلقك. اكتب المواقف أو الأفكار التي تثير قلقك في «سُلَّم القلق» لمعرفتها وقياس شدّتها.

**التعليمات**

١. دوِّن أكثر أسباب القلق شيوعاً لديك؛ قد تكون مواقف حقيقية أو أفكاراً.
٢. ضع كلّ موقف أو فكرة في الدرجة التي تمثّل شدّة القلق لديك (٠ = لا قلق، ١٠٠ = أقصى قلق). ضع أشدّ موقف في أعلى السلّم.
٣. إن كان لديك أمثلةٌ على الدرجة نفسها يمكنك كتابتها معاً.

**التعرّض التدريجي!**

حان وقت التطبيق العمليّ باستعمال سُلَّم القلق:

١. ابدأ بموقفٍ من الدرجات الأقل (٠–٤٠). إنْ لم تجد موقفاً منخفض القلق، ابتكر موقفاً مشابهاً أقلّ إزعاجاً.
٢. اكتب الموقف ودرجة القلق المتوقّعة.
٣. خطّط لتوقيت التعرّض وتخيّل خطواتك مسبقاً.
٤. أثناء التعرّض ابقَ حتى يهبط القلق بوضوح أو حتى تجمع معلومات كافية لتقدير الخطر الحقيقي. سجّل مستوى القلق مراراً.
٥. بعد الانتهاء، دوّن درجة القلق الفعلية؛ قد تكون أعلى أو أقلّ من المتوقّع.
٦. كرّر التعرّض نفسه في أقرب وقت، ويفضَّل في اليوم التالي. الهدف ملاحظة انخفاض القلق تدريجياً أو فهم الموقف على نحوٍ أعمق.
٧. عندما يهبط قلقك بوضوح انتقل إلى موقفٍ آخر. تذكّر أنّ النجاح لا يعني إنهاء السلّم في أسبوع؛ فمجرد تخفيف القلق في موقفٍ واحد إنجازٌ كبير.

تمت مراعاة اللغة العربية الفصحى المفهومة لدى السوريين، ودمج أمثالٍ شائعة وتلميحاتٍ إلى أهمية العائلة والصبر، مع الحفاظ على جوهر المحتوى العلاجي الأصلي.

**Stimulus 2: AI – Cognitive Restructuring**

**الأفكار التلقائية**

ليست المواقف بحد ذاتها هي التي تحدد مشاعرنا وسلوكياتنا، بل كيفية تفسيرنا لها. فقد يفهم أشخاص مختلفون نفس الموقف بطرق متباينة تماماً. وكما يقول المثل العربي: "العين بصيرة واليد قصيرة" - فما نراه ونفهمه قد يختلف عن .الواقع. هذه التفسيرات التي نقوم بها غالباً ما تحدث بشكل تلقائي وتنبع مما يُسمى في علم النفس بالأفكار التلقائية

**ما هي الأفكار التلقائية؟**

الأفكار التلقائية هي تلك الخواطر التي "تقفز" إلى أذهاننا دون تفكير مسبق. إنها تظهر بشكل انعكاسي أو اعتيادي، وذلك .على عكس الأفكار الواعية الموجهة التي نستخدمها عند التخطيط ليومنا أو التفكير فيما سنحضره لوجبة الغداء

بينما نقوم بهذا التخطيط الواعي، تمر في أذهان معظمنا سيل من الأفكار التلقائية. نحن لا نقرر التفكير بها، بل "تمر كالبرق" بشكل عفوي. قد تكون مثلاً: "لن أتمكن من إنجاز كل شيء"، "الحمد لله أنني أحضرت المال معي أمس"، أو "أين وضعت مفاتيح السيارة؟"

هذه الأفكار سريعة جداً وعابرة، وغالباً ما نكون أكثر وعياً بالمشاعر التي تثيرها من الأفكار نفسها. يمكن أن تتكون هذه :الأفكار من

- تفسيرات لمواقف مختلفة
- توقعات لما سيحدث
- ذكريات من أحداث سابقة، خاصة من مواقف مشابهة
- صور ذهنية متنوعة

**أنواع الأفكار التلقائية**

:يمكن أن تكون الأفكار إما محايدة أو إيجابية أو سلبية

**الأفكار المحايدة**: هي ملاحظات موضوعية دون أحكام قيمية. مثال: "هذه سيارة مثل سيارتي".

**الأفكار الإيجابية**: تساهم في تعزيز الشعور بالراحة والسعادة. مثال: "ما أجمل هذا اللقاء!" أو كما يقال: "القلب مرتاح والبال مستريح".

**الأفكار السلبية**: قد تكون انتقادات ذاتية أو أوامر داخلية أو حوارات نفسية، مثل: "لن أنجح في هذا أبداً"، "أبدو متوتراً جداً"، "لن أجد عملاً"، "لا أحد يرغب في الارتباط بي"، "لن أستطيع التعبير عما أريد"، "أنا عديم القيمة". في السياق العلاجي، تكتسب الأفكار السلبية أهمية خاصة لأنها غالباً ما تساهم في استمرار المعاناة النفسية.

نظراً لأن الأفكار التلقائية تحدث بشكل عفوي واعتيادي، قد يكون من الصعب إدراك أنها مجرد تفسيرات شخصية وليست حقائق مطلقة. وكما يقول المثل: "من كثر كلامه كثر خطؤه" - كذلك من كثرة تكرار فكرة ما لأنفسنا، نصدقها حتى لو لم تكن صحيحة.

**تمرين: ملاحظة الأفكار التلقائية**

لنفهم بشكل أفضل، إليك بعض الأمثلة المُكيّفة ثقافياً:

**مثال ١ - أحمد في المقهى:** يقف أحمد عند الكاشير في مقهى الجامعة ليدفع. يشعر بالتوتر الشديد من نظرات الطلاب خلفه ويلاحظ أن يديه بدأتا ترتجفان عند تسليم النقود. تمر في ذهنه هذه الأفكار التلقائية: "يا إلهي!"، "يا للإحراج!"، "يداي ترتجفان كأنني مريض"، "سأسقط النقود أمام الجميع". يشعر بالغضب والخيبة من نفسه ويغادر المكان.

**مثال ٢ - فاطمة في السوق:** تذهب فاطمة للتسوق استعداداً للعيد. السوق مزدحم جداً. تشعر بالضغط عندما تفكر في كل من يجب أن تشتري لهم هدايا. تظهر في ذهنها هذه الأفكار: "لن أتمكن من الشراء للجميع"، "لن أجد هدايا مناسبة". تشعر بتسارع ضربات قلبها ودوار خفيف. ثم تأتي الأفكار: "هناك خطب ما في قلبي"، "سأفقد الوعي وسط هذا الزحام. سيكون الأمر فظيعاً"، "أنا عاجزة عن فعل أي شيء".

في هذه الأمثلة، ليست المواقف بحد ذاتها هي المشكلة، بل أفكار أحمد وفاطمة وتفسيراتهما للموقف. الأفكار التلقائية غالباً ما تكون مختصرة وتظهر بشكل "مضغوط". فكرة مثل "هذا طبعي!" قد تعني: "فاتني الباص ولن يأتي آخر قبل ساعة!". وفكرة "يا ويلي!" قد تعني "كم أنا أخرق لإسقاطي كوب الشاي!".

يمكن أن تظهر هذه الأفكار كحديث صامت، أو كصور ذهنية، أو مزيج من الاثنين. لكن عادة ما يكون من السهل "ترجمتها" إلى كلمات عند التفكير فيها.

**اكتشاف الأفكار التلقائية**

قد يكون اكتشاف هذه الأفكار السريعة أصعب مما نتصور. طريقة فعالة للبدء في ملاحظة أفكارك السلبية التلقائية هي الانتباه لمشاعرك. عندما تشعر فجأة بمشاعر مزعجة أو صعبة، غالباً ما تكون قد مررت بفكرة سلبية تلقائية. لذا في المرة القادمة عندما تشعر بارتفاع القلق أو انخفاض المزاج - توقف لحظة، وفكر فيما مر بذهنك في الثواني السابقة.

وكما يقول المثل العربي: "من راقب الناس مات همّاً" - أحياناً مراقبة أفكارنا عن نظرة الآخرين لنا تسبب لنا معاناة لا داعي لها.

**تمرين: تسجيل الأفكار التلقائية**

الخطوة الأولى في تغيير هذه الأفكار هي ملاحظتها. أفضل طريقة لذلك هي تسجيلها مع المواقف التي تظهر فيها والمشاعر التي تثيرها.

للقيام بهذا التمرين، فكر في موقف حديث مررت به وكان صعباً وشعرت فيه بالضيق الشديد.

**كيفية ملء الجدول:**

1. صِف الموقف الذي مررت به في العمود الأول
2. ما الذي مر بذهنك في ذلك الموقف؟ ما هي الأفكار التي خطرت لك؟ اكتب جميع أفكارك في العمود الأوسط
3. ما هي المشاعر التي أثارتها هذه الأفكار؟ املأ العمود الثالث بالمشاعر التي شعرت بها

**مثال توضيحي:**

| **الأفكار التلقائية - جدول الأمثلة** | | |
| --- | --- | --- |
| **الموقف** | **الفكرة** | **الشعور** |
| تقديم عرض في المدرسة/العمل | "الجميع يرى أنني متوتر" "أنا ممل، لا أحد يريد ان يستمع الى" | خيبة أمل، قلق |
| الجلوس وحيداً في المنزل | كما يقول المثل: "اللي ما عنده قيمة ما حد يسال عنه." "انا احرجت نفسي حقا اليوم. انا عديم القيمة تماما" – لكن تذكر ان قيمتك لا تحدد بلحظة واحدة | حزن، اكتئاب |
| **ملاحظات ثقافية:**   - هذه الأفكار التلقائية شائعة وطبيعية، خاصة عند التأقلم مع بيئة جديدة - في ثقافتنا العربية، قد نشعر بضغط إضافي للحفاظ على صورة إيجابية أمام الآخرين - تذكر أن الأفكار ليست حقائق - إنها مجرد تفسيرات يمكن تغييرها | | |

تذكر: "الصبر مفتاح الفرج" - التدرب على ملاحظة أفكارك يحتاج وقتاً وصبراً، لكنه خطوة مهمة نحو فهم نفسك بشكل أفضل.

**Stimulus 3: Human - Exposure**

**القلق الشديد: منبه الخطر عند الجسم**

البعض من الناس يمكن أن يعاني من قلق قوي متكرر، قلق مستمر- أو نوبات قلق قصيرة تنتهي بسرعة.

يوجد هناك أمر واحد صحيح دائماً، أن القلق الشديد هو رد فعل من جسمك لشيء يبدو خطير. القلق الشديد هو نوع من الخوف! القلق هو أيضاً رد فعل غريزي للبقاء على قيد الحياة. في الأصل، من آلاف السنين الماضية كان القلق الشديد هو ما نجى من الحيوانات الخطيرة أو ساعدنا لإيجاد القوة للقتال! عندما نقابل نمر نصبح خائفين ونشعر بالقلق الشديد – بذلك يبدأ القلب بالخفقان بقوة، الجسم يرتجف والأفكار تعصف بشدة. هذه هو السبب في أن القلق قوي جداً – هو يجعل كل الجسم في حالة تأهب لمساعدتنا على القتال أو الهروب.

مازلنا حتى اليوم نشعر بنفس رد فعل القلق الشديد عندما يرتابنا الخوف. لا يهم ما إذا كان الخطر محدد كثيراً (مطارد من نمر، كدت أن تُدهس من باص) أو إذا كان الأمر بداخل رأسك ("ما حدا بدو يحكي معي"). الجسم لا يمكنه التمييز بين هذه المخاطر وإنما يستجيب دائماً بنفس الطريقة.

بغض النظر عن مدى قوة القلق، إلا أنه ليس خطيراً أبداً. القلق هو رد فعل غريزي للبقاء على قيد الحياة – ربما سيكون من التناقض قليلاً لو كان القلق خطيراً، أليس كذلك؟ نحن لا نقول إنه يجب عليك أن تكون شاكراً له، لكن بالحقيقة القلق هو ليس عدوك وإنما يريد لك الخير والبقاء على قيد الحياة.

**هكذا يمكنك تخفيف القلق الشديد**

في الواقع ليس الموقف بحد ذاته هو المشكلة الكبرى، وإنما ردة فعل القلق! هذا ما يجعلنا نهرب من الموقف.

حتى نخفف من قلقنا علينا أن نقوم تماما بعكس ما يقوله حدسنا – بمعنى أن نقف في الحدث بدلاً من الهروب منه. هذه الاستراتيجية بنيت على المعرفة، أن القلق الشديد غالباً ما ينتهي ويتلاشى لوحده إذا لم يهرب منه المرء.

**إذا قمت بالبقاء سيأخذ القلق وقتاً أطول لينخفض عما إذا غادرت فوراً، لكن هو ما يزال ينخفض!**

القلق غالباً ما يرتفع ثم ينخفض، نحن نسمي ذلك في العلاج المعرفي السلوكي "منحنى القلق". في الصورة بالأسفل قمنا برسم كيف يبدو منحنى القلق. عندما تمر بشيء مزعج وقلقك يزيد، يصعد بذلك المنحنى. لكن لا يمكن للمنحنى أن يصعد إلى ما لا نهاية. تذكر أن القلق لديه وظيفة لإبقائنا على قيد الحياة. في النهاية، غالباً ما يستوي المنحى كما في الصورة، *حتى عندما تبقى في الموقف!*

*
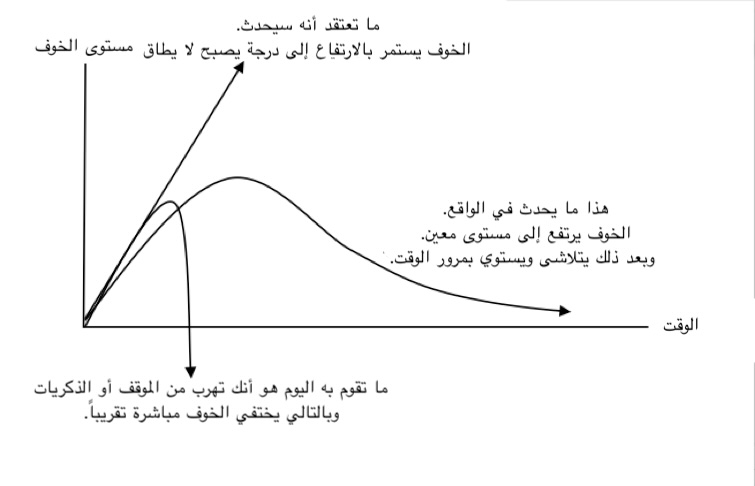
منحنى القلق:*

**حول التعرض**

**التعرض هو أحد أكثر الأساليب الفعالة في العلاج المعرفي السلوكي. هي تعمل بغض النظر ما إذا كنت خائف من الكلاب، العروض التقديمية، المستقبل، أو من الانهيار. هي تقوم بمساعدتك على التعود تدريجياً على ما يملئك بالقلق. هذا الأسلوب ليس لطيفاً، ولكنه يعمل بشكل جيد.**

- مهما كان القلق شديد فإنه دائماً ينخفض في النهاية - حقيقية فيزيولوجية
- التعرض هو التعود على القلق. هذا لن يجعل القلق يرتفع بنفس الشدة في المرة القادمة التي تمر بها بنفس القلق.
- أحياناً ينخفض القلق بسرعة أثناء التعرض، وأحياناً أخرى يأخذ الأمر بعض الوقت – لكن ذلك ينجح!
- إذا لاحظ الشخص أن القلق لا ينخفض بدرجة كبيرة يمكننا بدلاً عن ذلك التركيز على أن نتوقف في حالة القلق حتى نتعلم شيء جديد.
- إذا واجهت القلق الخاص بك بدرجة معتدلة، لمدة كافية ولعدة مرات، سوف لن يشكل قلقك أي عقبة لك في نهاية الأمر.

**تمرين: سلم القلق**

سوف نقوم باكتشاف المزيد عن القلق الخاص بك. أخبرنا المزيد عن قلقك من خلال كتابة ما يسبب لك القلق غالباً، التمرين اسمه "**سلم القلق**". هذا سيجعلنا نعرف ما الذي تقلق بشأنه عادةً وكم هي قوة قلقك.

**التعليمات**

1. اكتب أسبابك المعتادة للشعور بالقلق في القائمة. يمكن أن يكون ذلك مواقف واقعية، أو أفكار.
2. اكتب الفكرة أو الموقف في سطر يعكس جيداً مستوى القلق الذي يولده الموقف (الأرقام من اليمين تعكس مستوى القلق هناك 0 يعني لا قلق و 100 مزعج بشدة، لا يمكن أن يكون أسوء من ذلك! الموقف أو الفكرة الأكثر صعوبة على الإطلاق - سيكون مكانها في أعلى القائمة).
3. إذا كان لديك عدة أمثلة تسبب نفس مستوى القلق فمن الجيد أن تكتبهم جميعا في نفس السطر.

**التعرض!**

حان الوقت الآن لتجربة القيام بالتعرض للقلق! سلم القلق هو هنا لمساعدتك. المواقف أو الأفكار التي كتبتها في سلم القلق هي ما ستقوم بالتعرض له تدريجياً! أبدأ مع شيء يمكنك البدء بالتعرض له خلال هذا الأسبوع!

**هكذا تقوم بالتعرض:**

1. اختر شيء من قائمة سلم القلق الخاص بك والذي يوقظ قلقك بمستوى يتراوح بين (0 -40). إذا لم يكن لديك شيء منخفض القلق، حاول أن تبتكر بديل يشابه إلى حد ما كتبته، ولكن بدون أن يكون مليئا بالقلق حقاً.
2. اكتب ما قمت باختياره في المربع الأول هنا في الأسفل، ما هو مستوى قلقك المتوقع في المربع الثاني.
3. خطط لتوقيت مناسب لعمل التعرض. قبل البدء، بذهنك قم بمراجعة ما ستقوم به تماماً، حتى تكون مستعداً عندما يحدث ذلك!
4. التعرض: عندما يحين وقت التعرض فإن الهدف هو البقاء في الشعور المزعج حتى تشعر أن قلقك انخفض بدرجة واضحة أو حتى تشعر أنك حصلت على معلومات كافية حول الموقف لتقوم بعدها بتقييم مدى حقيقة خطورة الموقف! أثناء التعرض، قم بتقدير قلقك حتى تلاحظ أن القلق انخفض بدرجة واضحة مما كان عليه في أسوأ حالاته.
5. بعد التعرض – ارجع إلى هنا وقم بتعبئة المربع الثالث – كم شعرت بالقلق في الحقيقة من التعرض (يمكن أن يكون كلا الأمرين أكثر وأقل مما اعتقدته).
6. افعل مجدداً نفس أسلوب التعرض في أسرع وقت ممكن! إذا أمكن غداً. الهدف هو أن تشعر بعد عدة مرات من التعرض بدرجة أقل من القلق مقارنةً مع المرة الأولى، أو أن تتوقف بالموقف لمدة كافية حتى تستطيع تقييم مدى خطورة الموقف في الواقع.
7. عندما تشعر أن قلقك قد انخفض بدرجة واضحة يمكنك عندها البدء بالتعرض لشيء مختلف من سلم القلق الخاص بك! بالتأكيد ليس القصد هو أن تقوم بإكمال كامل القائمة خلال أسبوع. يمكنك اعتبار أن انخفاض القلق في موقف واحد هو نتيجة جيدة!

**Stimulus 4: Human – Cognitive Restructuring**

**الأفكار التلقائية**

ليس الموقف بذاته، وإنما كيف نفسر الموقف هو الأساس الذي يشكل كيف نشعر ونتصرف في موقف معين. بالتالي يمكن لأشخاص مختلفون أن يفهموا نفس الموقف بطرق مختلفة تماماً. التفسيرات التي نضعها تظهر غالباً كعادة وأصلها كما نطلق عليها عادةً في لغة علم النفس "الأفكار التلقائية".

الأفكار التلقائية هي أفكار “تقفز أمامنا” فجأة بدون أن نفكر فيها. هي فقط موجودة هناك بحكم العادة أو كمنعكس. على عكس الأفكار الواعية التي تتحكم بها عندما تخطط ما ستقوم به أثناء اليوم أو ما ستشتريه للعشاء مثلاً... بينما نقوم بهذا التخطيط الواعي يكون لدى معظمنا احتمال كبير لظهور الكثير من هذه الأفكار التلقائية في عقلنا. نحن نقرر ألا نفكر فيهم بالرغم من معرفتنا أنهم يتجولون في أذهاننا بشكل عفوي. على سبيل المثال يمكن أن تكون أفكار مثل "*أنا ما رح ألحق"، "من حظي إني سحبت المصاري مبارح" أو "يا ترى وين مفاتيح السيارة؟"*

الأفكار التلقائية سريعة ومتقلبة جداً ونحن عادةً واعيين للشعور الذي يأتي بعد الفكرة أكثر من الفكرة نفسها. الأفكار تكون عبارة عن:

- تفسيرات من مواقف مختلفة
- تنبؤات لما يمكن أن يحدث
- ذكرى من حدث سابق، مثلاً من مواقف مشابهة
- صور من أمور متعددة

الأفكار يمكنها أن تكون إما حيادية، إيجابية أو سلبية.

الأفكار الحيادية هي أفكار لا تحمل أي نوع من القيم وإنما تتعلق بشيء موضوعي واضح محدد. مثال: "*هناك سيارة تشبه سيارتي*". الأفكار الإيجابية التلقائية تساهم بتقوية الخبرات التي تعطينا شعور جيد. مثال: *"كم كان ذلك ممتعاً*"

الأفكار التلقائية السلبية ممكن تعمل كنداءات انتقادية، مناشدات أو حوارات داخلية، على سبيل المثال: *"هالشي عنجد سيء. قديش بينت متوتر!" "ما رح أحصل على شغل أبداً"، "ما حدا بدو يكون معي"، "ما رح أقدر أعبر عن شو بدي أحكي"، "أنا نكرة*". في السياق العلاجي، تعتبر الأفكار التلقائية السلبية مهمة بشكل خاص، بسبب مساهمتها غالباً على استمرار مشاكل الصحة النفسية.

لأن الأفكار التلقائية تظهر بشكل عادي وعفوي يمكن أن يكون من الصعب الاعتقاد أن الأمر متعلق بتفسيرات شخصية وليس حقائق فعلية. كلما تكرر شيء معين لنفسك، كلما يزيد احتمال أن تصدق هذا الشيء أكثر.

**تمرين: تسجيل الأفكار التلقائية**

حتى الآن قمنا بمحاولة وصف ما يميز الأفكار التلقائية. ما سيأتي تالياً هو عدة أمثلة لمساعدتك على فهم أي نوع من الأفكار نقصد.

مروان يقف أول طابور الدفع عند موظف الحساب بمقهى الجامعة ويريد أن يدفع الحساب. هو يشعر بالتوتر الشديد لكون كل النظرات تتجه نحوه ويشعر كيف أصبحت يديه ترتجف عندما أراد أن يناول النقود. ما يلي أفكار تلقائية خطرت على باله: *"ييييي لااا!"، "شو هالإحراج"، "شكلي عم أرجف مثل المدمن"، "رح أوقع كل المصاري*". هو شعر بالغضب وخاب أمله بنفسه وترك المكان.

نور ستتسوق لعيد الميلاد من منتصف كانون الأول. إنه أسبوع الراتب والمتاجر مزدحمة. هي بدأت تشعر بتوتر عندما فكرت بكل الأشخاص الذين سوف تقوم بشراء هدايا لهم. ما يلي أفكار تلقائية ظهرت عندها: "*ما بلحق أشتري هدايا للكل"، "ما رح لاقي شغلات مرتبة"*. نور شعرت بضربات قلبها تدق بسرعة وبدأت تشعر بدوخة. هنا حضرت الأفكار: "*في شيء غلط بقلبي*"، "*رح أغيب عن الوعي بين كل هالناس، ياااه على الإحراج*!", "*أنا ما بعرف أعمل بشيء*".

في الأمثلة السابقة ليس المهم ما تصفه المواقف بحد ذاتها وإنما أفكار مروان ونور وتفسيراتهم لتلك المواقف. الأفكار التلقائية عادةً هي مضغوطة وتظهر "*بصيغة مختصرة*". الفكرة "*كالعادة!*" يمكن أن تعني مثلاً: "*هلىء راح القطار ولازم استنى 50 دقيقة للقطار التالي!*". الفكرة الأخرى "*يييي لااا*" يمكن أن تعني "*يا لغبائي لأني وقعت علبة الحليب!".* على أي حال غالباً ما يسمح "ب*إخراج*" أو "*طي*" الفكرة إذا أردنا أن نفهم حقيقة ما يعنيه الشخص.

الأفكار تأتي أحياناً همس مثل (الوشوشة). يمكن أن تأتي أيضاً على شكل صور، أو بشكل "مختلط" (كِلا الأمرين همس وصور بنفس الوقت). مع ذلك غالباً ما يكون من الأسهل ترجمتهم إلى "نص". صورة عن نفسي وأنا أنشف الحليب المسكوب على الأرض يمكن أن تظهر جيداً برأسي مثل الفكرة *" يييي لااا*". وبالمثل كما لو أن الفكرة " *يا لغبائي لأني أوقعت علبة الحليب*!" يمكن أن تظهر فجأة. النقطة المهمة هي أننا غالباً ما نعرف ما نقصده بالرغم من ظهورها على شكل صور أو رد فعل عفوي.

يمكن أن يكون اكتشاف هذه الأفكار التلقائية السريعة جداً أصعب مما يتخيله المرء في البداية. لكن مساعدة جيدة وطريقة فعالة لبدء ملاحظة أفكارك التلقائية السلبية، هي أن تكون واعياً لمشاعرك. عندما تشعر فجأةً بالضيق أو بشعور مزعج، غالباً ما تكون قد خطرت لك فكرة تلقائية سلبية. لذلك عند مواصلتك الشعور بأن القلق يزداد أو الحالة المزاجية تنخفض – توقف قليلاً، وفكر بما خطر على بالك باللحظات السابقة.

*تمرين: سجل الأفكار التلقائية*

الخطوة الأولى في إمكانية تغيير هذه الأفكار، هي أن تقوم بملاحظتهم فقط. وأفضل طريقة لذلك هو القيام بتسجيلهم وكتابتهم سوياً مع المواقف التي تأتي فيها بالإضافة للمشاعر التي تؤدي إليها.

حتى تقوم بملء التمرين عليك أن تفكر بموقف ما تعرضت له مؤخراً، موقف اعتبرته صعب وشعرت بشكل سيئ بسببه.

انظر إلى المثال حتى تفهم كيف يمكنك ملء القائمة، بعد ذلك جرب من الموقف الخاص بك الذي اخترته!

1. أوصف الموقف الذي مررت به في الأسفل إلى اليمين.
2. ما الذي خطر على بالك في الموقف، أي الأفكار فكرت فيها؟ اكتب كل أفكارك في المنتصف! أحيانا ما تكون فكرة واحدة فقط، أو صورة، أو فكرة واحدة تولد العديد من الأفكار الأخرى. اكتب ما خطر لك أو ما رأيته أمامك!
3. ما المشاعر التي قادت إليها أفكارك؟ املأ العمود الثالث بما اختبرته من مشاعر. هذا الأمر صعب، لذلك افعل أفضل ما تستطيع!

مثال:

| **شعور** | **فكرة** | **مثال: موقف** |
| --- | --- | --- |
| قلق ، خيبة أمل (يائس) | "أنا ممل، ما في حدا بدو يسمعني"  "الكل شايف إني متوتر!" | تقديم وظيفة في المدرسة |
| حزين، مغموم | "اليوم أحرجت حالي عنجد. قديشني عديم القيمة وتافه" | أجلس وحيد في المنزل |
