# Supplementary figures and images for: Cultural Relevance and Acceptability of Cognitive Behavioral Therapy Techniques Adapted by AI or a Human Psychologist: Experimental Study
Source: JMIR Form Res. 2026 May 4;10:e91056. doi: 10.2196/91056 (PMC13138788; doi:10.2196/91056)

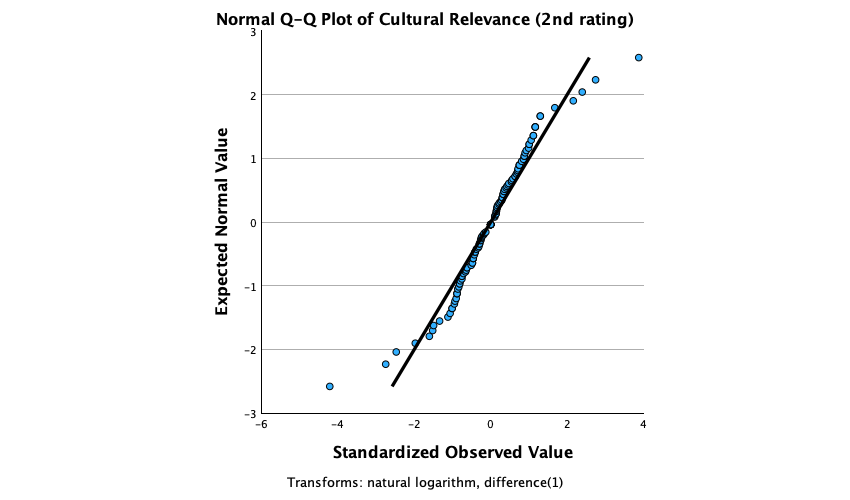
**Appendix 5: Q-Q Plots**


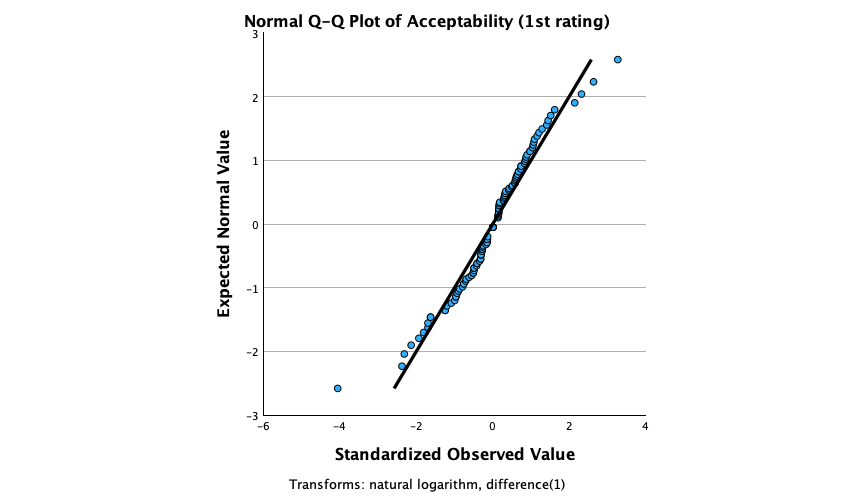


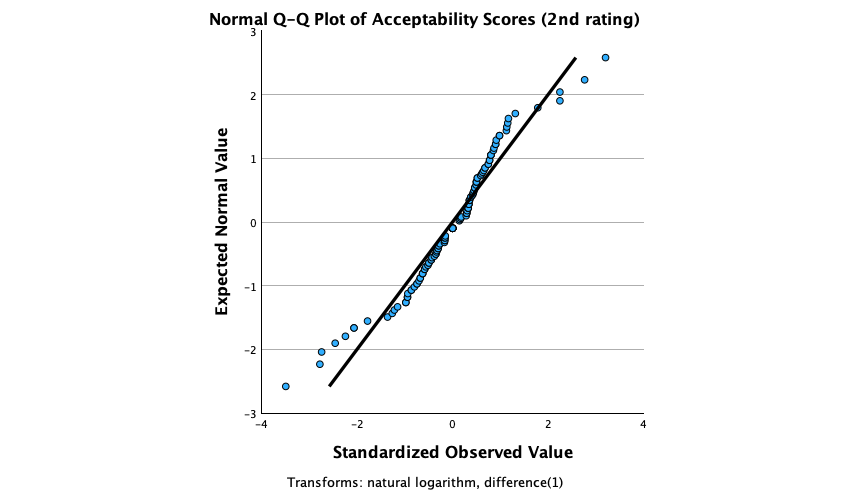

Supplement: Multimedia Appendix 5 [file formative-v10-e91056-s005.docx]
